# Population structure and genetic diversity of a germplasm for hybrid breeding in rye (*Secale cereale* L.) using high-density DArTseq-based silicoDArT and SNP markers

**DOI:** 10.1007/s13353-022-00740-w

**Published:** 2023-01-03

**Authors:** Agnieszka Niedziela, Piotr Tomasz Bednarek

**Affiliations:** Plant Breeding and Acclimatization Institute - National Research Institute, 05-870 Błonie, Radzików, Poland

**Keywords:** Rye, Genetic diversity, CMS Pampa, Hybrid breeding

## Abstract

Investigating genetic structure and diversity is crucial for the rye hybrid breeding strategy, leading to improved plant productivity and adaptation. The present study elucidated the population structure and genetic diversity of 188 rye accessions, comprising 94 pollen fertility restoration lines (RF) and 94 cytoplasmic male-sterile (CMS) lines with Pampa sterilizing cytoplasm using SNP and silicoDArT markers from the diversity array technology (DArT)-based sequencing platform (DArTseq). Expected heterozygosity (*H*_*e*_) and Shanon’s diversity (*I*) indexes varied slightly between marker systems and groups of germplasms (*H*_*e*_ = 0.34, *I* = 0.51 for RF and CMS lines genotyped using SNPs; *H*_*e*_ = 0.31, *I* = 0.48, and *H*_*e*_ = 0.35, *I* = 0.53 for RF and CMS using silicoDArTs, respectively). ANOVA indicated moderate variation (7%) between RF and CMS breeding materials. The same parameter varied when chromosome-assigned markers were used and ranged from 5.8% for 5R to 7.4% for 4R. However, when silicoDArT markers were applied, the respective values varied from 6.4% (1R) to 8.2% (3R and 4R). The model-based (Bayesian) population structure analysis based on the total marker pool identified two major subpopulations for the studied rye germplasm. The first one (P1) encompasses 93 RF accessions, and the second one (P2) encompasses 94 CMS and one RF accession. However, a similar analysis related to markers assigned to selected chromosomes failed to put plant materials into any of the populations in the same way as the total marker pool. Furthermore, the differences in grouping depended on marker types used for analysis.

**Supplementary Information:**

The online version contains supplementary material available at 10.1007/s13353-022-00740-w.

## Introduction

In rye breeding projects, it is crucial to make high-yielding cultivars resistant to diseases, lodging, and sprouting. Grain output and quality might vary greatly depending on cultivars (Arseniuk and Oleksiak 2003; Hansen et al. 2004; Bujak et al. 2006; Dynkowska et al. 2015; Alijošius et al. 2016; Iwańska et al. 2020). Hybrid rye cultivars have been shown to produce 20–25% more grain than the best population cultivars while maintaining grain quality (Arseniuk and Oleksiak 2003; Laidig et al. 2017; Linina et al. 2019). The heterosis phenomenon causes this beneficial impact.

According to Fisher et al. (2010), heterosis is stronger when the parent forms come from different genetic populations. The populations are called heterotic groups. A group of related or unrelated genotypes from the same or other populations that exhibit comparable combining capacity and heterotic response when crossed with genotypes from other genetically dissimilar germplasm groups is referred to as a “heterotic group” (Melchinger and Gumber 1998). So, Reif et al. (2003) say that it depends on the available materials to determine the genetic makeup and/or genetic diversity of heterotic groupings. Regardless of the year and location of the experiment, some research showed that the larger the genetic distance between parental plants, the more significant the heterosis effect for most of the observed features (Reif et al. 2003; Liersch et al. 2010; Tomkowiak et al. 2019, 2020). For example, in studies of maize (Becker and Link, 2002; Betrán et al. 2003; Zhang et al. 2017a, b; Tomkowiak et al. 2019, 2020) and rapeseed (Becker and Engqvist 1995; Liersch et al. 2010), researchers found a link between the heterosis effect on seed yield and the genetic distance between the parents.

However, the genetic distance coefficients employed for germplasm analysis affect how genetic pools form (Reif et al. 2003; Tomkowiak et al. 2020). Additionally, the nature of the marker system may be significant (Frisch et al. 2010; Tomkowiak et al. 2020). Tomkowiak et al. (2020) found that AFLP and RAPD markers were less valuable than SSR, SNP, and silicoDArTs when choosing parental components for crossing maize. Frisch et al. (2010) recommended a different solution based on transcriptome information. The author showed that general estimates of hybrid performance based on field data or older models with DNA markers are less accurate than distances based on the transcriptome.

It should be emphasized that predicting the consequences of heterosis is not always possible by relying solely on genetic groups derived using similarity indices based on molecular markers (Kaeppler 2012). Wheat or durum wheat, for instance, does not demonstrate a relationship between genetic distance and heterosis (Martin et al. 1995; Barbosa-Neto et al. 1996; Perenzin et al. 1998; Corbellini et al. 2002; Dreisigacker et al. 2005; Krystkowiak et al. 2009). That could be because the species lacks genetic diversity or because the studies only used a small amount of parental germplasm, making it hard to find links between genetic distance and heterosis. On the other hand, parental forms of sunflower hybrids (Gvozdenović et al. 2009), waxy and sweet corn (Dermail et al. 2020), and oilseed rape (Yu et al. 2005) showed an important link between genetic distance and heterosis impact. Also, Fischer (2010), Zhang et al. (2017a, b), and Boyaci et al. (2020) found that using heterotic pools to make hybrids by mixing the tester with each trait could lead to better results.

Rye hybrid breeding programs are a prime example of using heterotic gene pools for economic gain (Geiger and Miedaner 1996). In Germany, two heterotic genetic pools that represented the maternal (“Petkus” pool) and paternal (“Carsten” pool) genotypes were selected (Geiger and Miedaner 1996). Plants from both gene pools were selfed repeatedly to make the parental inbred rye lines (Fischer 2010). In the breeding process, inbred lines were primarily generated from crosses between elite inbreeds within heterotic pools, reducing genetic diversity (Duvick et al. 2004). Alternatives are being sought since using the breeding strategy to discover heterotic pools based on, for example, combining ability is relatively laborious. Furthermore, some species or materials might not have heterotic groups.

Numerous population studies have been carried out on the rye germplasm. DNA-based molecular marker techniques such as simple sequence repeats (SSR), amplification fragment length polymorphism (AFLP), random amplification polymorphic DNA (RAPD), diversity array technology (DArT), and single nucleotide polymorphism (SNP) were used to measure the genetic diversity (Ćwiklińska et al. 2010; Myśków et al. 2010; Chikmawati et al. 2012; Bolibok-Brągoszewska et al. 2014; Targońska et al. 2016; Sidhu et al. 2019; Vendelbo et al. 2020; Targońska-Karasek et al. 2020). The findings suggest that the genetic diversity of the *Secale* genus is greater than that of landraces or even cultivated accessions (Shang et al. 2006; Bolibok-Brągoszewska et al. 2014). Genetic differences (*Phi*_*PT*_) based on DArT markers range from 0.15 to 0.20 between landraces, breeding materials, varieties from the PAS BG seed bank in Warsaw-Powsin (Poland), and accessions from professor A. Łukaszewski’s collection (Bolibok-Brgoszewska et al. 2014). The same authors found no difference when comparing landraces, cultivated materials, and varieties from PAS BG (*Phi*_*PT*_ below 0.05). On the other hand, restorer lines (R) from the “Petkus” and “Carsten” gene pools were genetically distinct (*F*_*ST*_ = 0.332) from non-restorer germplasm (NRG) combined with cytoplasmic male-sterile (CMS) lines based on the “Gülzow” (G) type of cytoplasm, according to a study by Vendelbo et al. (2020) using SNP markers. Hierarchical clustering and principal component analysis showed that the seed parent (NRG&CMS) and pollen parent (R) populations were genetically different. Small populations of the identical seed and pollen parent pools from the “Petkus” and “Carsten” showed a significant difference (*F*_*ST*_ = 0.229) when Bauer et al. (2017) employed markers from a 600 K high-density SNP array. This trend was supported by a principal coordinate analysis (PCoA) that showed a clear difference between the parent populations. A similar study was not done on the maternal and restorer lines used by Polish breeding companies, which is unfortunate. However, rye hybrid breeding companies have extensive expertise in assessing plant material. Additionally, different companies preferred breeding maternal and parental lines. This makes it more likely that genetically different pools have formed. Knowing this could help assess heterogenic pools in the rye.

We hypothesize that the choice of a DNA-based marker system might be crucial for the identification of putative pools, and that due to breeding pressure, the markers assigned to the chromosomes under such pressure would be more effective in differentiating materials than the entire marker set.

## Material and methods

### Plant material and DNA extraction

For the investigation, a total of 188 elite hybrid breeding components of rye (*Secale cereale* L.) were chosen, including 94 restorer lines (RF) and 94 cytoplasmic male-sterile (CMS) lines with Pampa sterilizing cytoplasm (Table S1). For RF and CMS-based lines, the inbreeding stage ranged from F3 to F11 and F2 to F3 generations, respectively. The plants were assisted by DANKO Plant Breeding Ltd., located in Choryń 27, 64–000 Kościan, Poland. All restorers and 83 CMS lines originated from the Breeding Department of Choryń and 11 CMS lines from the Breeding Department of Laski.

Total DNA samples were extracted from fresh leaf tissue of three plants (equal parts) representing the given line using a DNeasy Plant Mini Kit 250 according to the manufacturer’s instructions. The quality of the isolated DNA was measured on a 1% agarose gel and with a NanoDrop ND-1000 Spectrophotometer (Thermo Fisher Scientific, Waltham, MA, USA).

### Genotyping

DNA samples were processed for genotyping using DArTseq™ technology offered by Diversity Arrays Technology (Pty) Ltd. in Canberra, Australia. DArTseq is a genotyping-by-sequencing system that sequences the most informative representations of genomic DNA samples using next-generation sequencing platforms (HiSeq 2500 in our case). The protocol has been described previously in detail by Kilian et al. (2012) and Melville et al. (2017). The results consist of two marker types: SNP (codominant) and silicoDArT (dominant). SNP markers were coded in a binary matrix. Each locus was represented by two consecutive lines to preserve its codominant nature. The presence of an SNP relative to the reference sequence was denoted as 1, while the absence was 0. So, the array showed homozygotes as 1/1 or 0/0 and heterozygotes as 1/0. SilicoDArT markers were coded in a binary fashion according to their absence (0) or presence (1) in genomic representations. The markers were filtered for reproducibility ≥ 0.95 and CallRate ≥ 0.90. Furthermore, missing data (< 10%), and minor allele frequency < 5% were discarded for future analysis.

Marker position on the rye chromosomes was defined by diversity arrays technology (Pty) Ltd. according to the position(s) on contig(s) with the best alignment of marker/tag to the “Rye_v2” model genome. The values reflect distance as the number of the base pair from the beginning of the chromosome.

### Genetic diversity and population structure

Primary genetic diversity indices were calculated using the GenAlEx v.6.5 Excel add-in (Peakall and Smouse 2012). These included the percentage of polymorphic markers (PPL), the number of different alleles (*N*_*a*_), the number of effective alleles (*N*_*e*_), the number of private alleles/bands, Shannon's information index (*I*), expected heterozygosity (*H*_*e*_), unbiased expected heterozygosity (*uH*_*e*_), observed heterozygosity (*H*_*o*_), and the fixation index (*F*).

For the evaluation of polymorphic information content (PIC) of dominant bi-allelic silicoDArT markers, the following formula was used: PIC = 1 − (*p*^2^ + *q*^2^), where “p” is the frequency of present alleles and “q” is the frequency of null alleles (Serrote et al. 2000). Botstein et al. (1980) came up with a formula to figure out the PIC for codominant (SNP) markers:

$$PIC= 1-\sum_{i=1}^{n}{p}_{i}^{2}$$

In this equation, *n* is the number of alleles, and *p*_*i*_ and *p*_*j*_ are the allele frequencies in populations *i* and *j*, respectively. PIC values range from 0 (monomorphic) to 0.5 (highly informative, where many alleles have the same frequency). Results were given for the total number of markers searched in the RF and CMS lines and for the markers assigned to each rye chromosome separately.

The GeneAlex Excel add-in software (Peakall and Smouse, 2012) used the analysis of molecular variance (AMOVA) to divide molecular variance into two groups: (1) between RF and CMS breeding materials and (2) within RF and CMS breeding materials. The pairwise *F*_*ST*_ (codominant SNP markers) or *Phi*_*PT*_ (dominant silicoDArT markers) coefficients and Nei’s minimum genetic distance (Nei 1972) between the RF and CMS lines were calculated in GeneAlex. The *Phi*_*PT*_*/F*_*ST*_ values were calculated to evaluate within-population variance and population differentiation. The estimated probability values from 1000 permutations were used to figure out if the way the variance components were split up was significant. The analysis was done using the total number of markers and the markers assigned to each chromosome.

The dartR package was used to look at a pairwise genetic distance (GD) matrix of codominant SNP markers. The dissimilarity between the two sets was measured using Jaccard’s distance. GD matrices were calculated for each of the rye chromosomes. A pairwise genetic similarity (GS) matrix (Jaccard coefficients) (Jaccard 1908) based on molecular markers assigned to the given chromosome (and total marker pool) and utilizing dominant silicoDArT markers was calculated in PAST software, Version 4.06b (Hammer et al. 2001). For the analysis, ten hundred markers with the smallest amount of missing data per individual (less than 4.5%) were applied to fulfill PAST requirements. GS values were converted into GD by subtracting GS from 1. In PAST software, a violin plot was used to show how the GS values for all markers and chromosomes were spread out in terms of density. The genetic distance matrices were compared using the Mantel test (Mantel 1967) in the GenAlex Excel add-in software. The comparison led to correlation and determination coefficients.

In the XlStat software (XlStat 2019), a one-way analysis of variance (ANOVA) with Tukey’s pairwise comparisons was used to look for differences between the GD means of different chromosomes.

The putative structure among all 188 rye accessions genotyped with SNP and silicoDArT markers was tested using principal component analysis (PCA) based on the Jaccard distance matrices in PAST software, Version 4.06b (Hammer et al. 2001). Next, Bayesian analysis with Structure 2.2.3 (Pritchard et al. 2000b) was used to figure out how many populations (K) could capture the main structure in the data. The admixture model, a burn-in period of 50,000 MCMC iterations, and 100,000 run lengths were used in the analysis. There were eight separate runs for every simulated value of *K*, from 1 to 5. The most likely *K*-value was determined by structure harvester (Earl and Vonholdt 2012) using the log probability of the data [LnP(D)] and delta K (ΔK) based on the rate of change in [LnP(D)] between successive *K*-values (Evanno et al. 2005). Next, the average genetic structure was estimated using the CLUMPP software (Jakobsson and Rosenberg 2007). With the Distruct software (Rosenberg, 2004), a bar graph of the structure of the population was made. The analysis was done separately for the total number of SNP and silicoDArT markers and markers assigned to the given chromosomes.

## Results

### Genetic diversity

In total, 188,150 (62,580 SNP and 125,780 silicoDArT) signals were generated for 188 rye accessions using DArTseq technology. After filtering, there were 57,550 markers (14,300 SNP and 43,220 silicoDArT).

Percentages of polymorphic markers (*PPL*) for investigated materials equaled 99% (SNP) and 100% (silicoDArTs). The genetic variation parameters calculated for SNP markers, such as observed heterozygosity (*H*_*o*_), the diversity index (*H*_*e*_), and fixation index (*F*) for the entire set of accessions, had values of 0.074, 0.342, and 0.786 for RF and 0.125, 0.338, and 0.634 for CMS, respectively. Shannon’s information index (*I*) was 0.51 for both the RF and CMS lines. There were 226 (RF) and 240 (CMS) private alleles (PrA) among SNP markers (Table 1).

**Table 1:** The mean of different genetic parameters, including the number of different alleles (*Na*), number of effective alleles (*Ne*), Shannon’s information index (*I*), number of private alleles (SSR)/bands (silicoDArT) (PrA/B), observed heterozygosity (*H*_*o*_), expected heterozygosity (*H*_*e*_), unbiased expected heterozygosity (*uH*_*e*_), fixation index (*F*), and percentage of polymorphic loci (PPL) in RF and CMS parental components used for hybrid breeding in Poland

Type of the markers	lines	*N*_*a*_	*N*_*e*_	*I*	PrA/B	*H*_o_*	*H*_*e*_	*uH*_*e*_	*PPL*	F*
SNP	RF	1.99	1.583	0.513	226	0.074	0.342	0.345	99.0	0.786
	CMS	1.99	1.576	0.509	240	0.125	0.338	0.341	99.1	0.634
silicoDArT	RF	2.00	1.452	0.484	3	-	0.313	0.315	99.9	-
	CMS	2.00	1.522	0.525	17	-	0.348	0.349	99.9	-

^*^For SNP only (Ho is available for co-dominant markers only)

The analysis omits a single restorer line which was classified with CMS lines according to the structure results

The genetic variation parameters calculated for silicoDArTs were much lower for restorers (*H*_*e*_ = 0.313, *I* = 0.484) than for cytoplasmic male-sterile lines (*H*_*e*_ = 0.348, *I* = 0.525). In the RF and CMS materials, there were three and seventeen private bands (PrB) among the silicoDArTs.

**Fig. 1:**
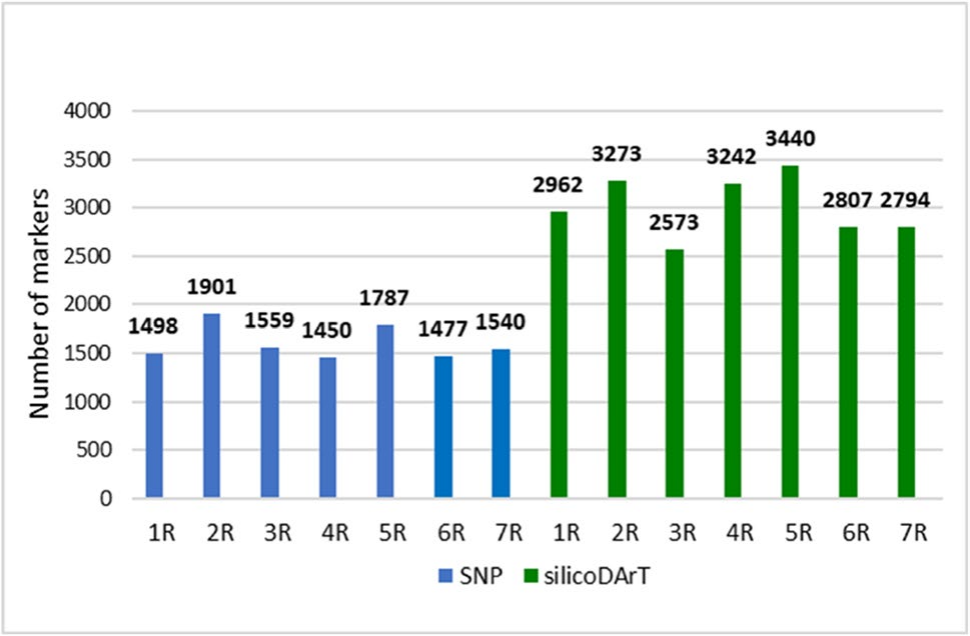
Distribution of SNP and silicoDArT makers across all chromosomes in the rye genome

There were 1498, 1901, 1559, 1450, 1787, 1477, and 1540 SNP markers on the chromosomes 1R, 2R, 3R, 4R, 5R, 6R, and 7R, respectively, in the RF and CMS materials that were studied (Fig. 1). The chromosomal location of the 3088 markers remains unknown. In the case of silicoDArTs, 2962 (1R), 3273 (2R), 2573 (3R), 3242 (4R), 3440 (5R), 2807 (6R), and 2794 (7R) were assigned to the particular rye chromosomes (Fig. 1), and 22,165 remained unassigned (NA). The markers (SNPs and silicoDArTs) were evenly distributed along the rye chromosomes. On average, when SNPs were used, markers were separated by a distance ranging from 26.1 to 35.1 bp in the case of the 2R and 1R chromosomes. When silicoDArTs were used, similar values varied from 14.5 to 23.1 bp for the 5R and 6R chromosomes, respectively. It should be stressed, however, that the distal arms were poorly saturated with markers. In those regions, only 1 to 2% of markers were available. If those gaps were considered, the average marker distribution ranged from 33.7 bp (2R) to 56 bp (7R) for SNPs and from 21.5 bp (5R) to 43.9 bp (1R) for silicoDArTs. The most extended gaps were found on the 7R (8160 bp for SNPs) and 1R (29,631 bp for silicoDArTs) chromosomes.

**Fig. 2:**
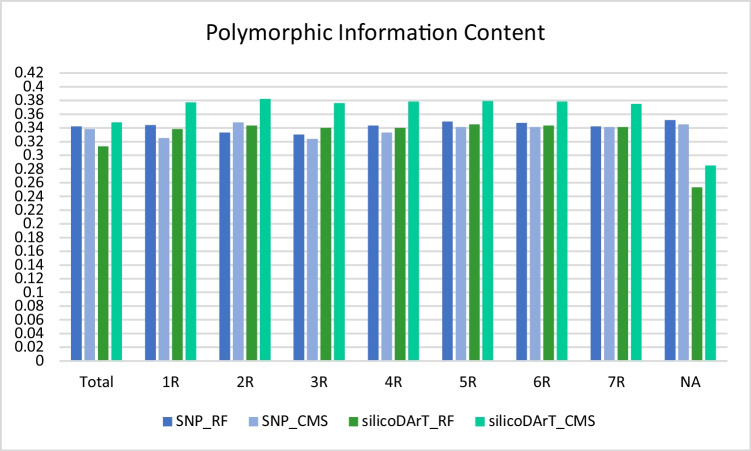
Average polymorphic information content (PIC) values of SNP and silicoDArT markers calculated for the entire genome (Total) and particular chromosomes (1R-7R) of restorer (RF) and cytoplasmic male-sterile (CMS) lines. NA, markers not assigned to any chromosome

The average PIC value for all analyzed SNP markers was 0.34 for both the RF and CMS lines. A very high PIC value (0.4–0.5) was observed for about 44.3% of the markers. As many as 6.2% (RF) and 7.6% (CMS) SNP markers with a PIC value of > 0.1 were present. When the markers were put on chromosomes, markers on 2R (CMS), 5R (RF), and 6R (RF) had a slightly higher average PIC (0.35 vs. 0.33–0.34) (Fig. 2).

In the silicoDArT markers, the PIC values varied for RF and CMS lines and reached 0.31 and 0.35, respectively. In total, 51.5% (RF) and 36.6% (CMS) of them were characterized by a PIC value between 0.4 and 0.5, whereas only 2.9% (RF) and 1.5% (CMS) had a PIC value ranging from 0 to 0.1. The average PIC values were comparable for all RF (0.34) and CMS (0.38) chromosomes. However, they varied significantly for not-assigned markers (0.25 for RF and 0.28 for CMS lines) (Fig. 2).

**Fig. 3:**
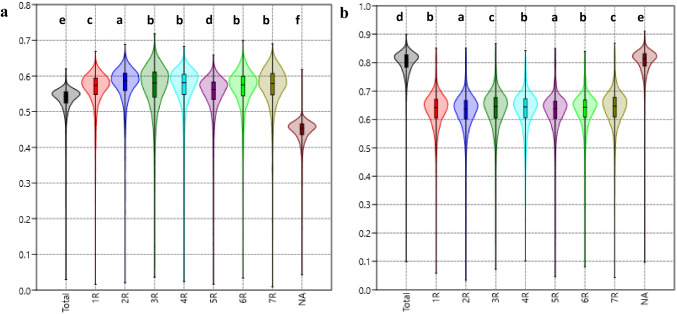
Violin plots show the density distribution between plant materials evaluated based on genetic distance (GD) values calculated with SNP (**a**) and silicoDArT (**b**) markers. The abscisic axis displays data based on markers allocated to all rye chromosomes, markers assigned to individual chromosomes, and markers that have not yet been assigned to any chromosome. The ordinate axis displays genetic distance. The violin plots illustrate the range of genetic distances across plant materials. Vertical bars reflect the genetic distances between studied materials lying within the most typical range, whereas horizontal lines inside the box shown mean values. The letters at the top of each plot show how the Tukey’s HSD mean comparison test with a *p*-value of 0.0001 grouped the data

The average GD among rye genotypes calculated with a total number of SNP markers ranged from 0.030 (“L1909”– “L1961”) to 0.619 (“SE58P/12”– “SO79R”) and reached an average value of 0.535. The average GD value differentiated slightly but significantly (*p* < 0.0001) across the chromosomes and reached 0.562, 0.575, 0.567, 0.567, 0551, 0.565, and 0.566 for 1R, 2R, 3R, 4R, 5R, 6R, and 7R, respectively (Fig. 3a). When the plants were looked at with markers that did not belong to any of the chromosomes, the mean GD value was 0.452.

The average genetic distance (GD) calculated using Jaccard’s coefficient for rye breeding materials using a total number of silicoDArT markers was 0.799 and varied from the lowest of 0.098, which occurred between RF line “L1909” and “L1961,” to the highest of 0.991, which occurred between RF “L1995” and CMS lines: “S195P/18” (0.897), “S246P/18” (0.897), “S235P/18” (0,896), “LS379P/18” (0.895), “S46P/18” (0,892), “S222P/18” (0.892), “LS483P/18” (0,891), “S29P/18” (0.891) and “S211P/18” (0.890). RF “L1995” reached the highest values of GD (> 0.850) for all CMS lines. The average GD values across the chromosomes were 0.633, 0.629, 0.635, 0.633, 0.630, 0.633, and 0.637 for 1R, 2R, 3R, 4R, 5R, 6R, and 7R, respectively (Fig. 3b). The highest mean value of GD (0.803) was determined when markers that did not correspond to any of the chromosomes were used.

The lowest genetic distance between RF lines “L1909” and “L1961” was noticed independently of the marker types and the chromosomes for which the matrices were calculated. The highest GD depends on marker types and chromosomes. Maximum SNP values range from 0.659 (5R) to 0.718 (3R), while maximum silicoDArT values range from 0.843 (4R) to 0.866 (7R). The RF and CMS lines with the highest GD values were different depending on the chromosome. However, in silicoDArTs, the same RF line, “L1995,” always showed up in such a pair with the earlier-mentioned CMS lines, no matter the chromosome.

Comparing the genetic distance, matrices for all of the RF and CMS materials that were analyzed, evaluated using SNP or silicoDArTs markers, and assigned to the rye chromosomes revealed (Fig. 3) that the GD between plant materials was higher when calculated using SNPs as opposed to silicoDArTs for all of the analyzed chromosomes. When GD matrices for the total number of markers and those for markers not assigned to any chromosomes were compared, the relative density distribution values for SNPs were lower than for silicoDArTs. Also, the data from the SNP markers assigned to a chromosome showed less separation between plant materials than the GD matrices made from the entire pool of markers and those not assigned to any chromosome. The silicoDArTs showed the opposite behavior. It should be noted that several materials had a significant GD distance. But the mean GD values for materials based on SNPs or silicoDArTs and assigned to chromosomes were closer. The violin charts illustrate the problem.

The analysis of molecular variance for all GD matrices using various marker types showed that the variations in mean GD values were significant (SNP: *F* = 4969.5, *p* < 0.0001; silicoDArT: *F* = 22,952.9, *p* < 0.0001). When SNP-based matrices were considered, the chromosomes 3R, 4R, 6R, and 7R formed one group, whereas the rest formed the other group. Additionally, the total and NA markers formed distinct groups. The silicoDArT-based matrices revealed that 1R, 4R, and 6R; 2R and 5R; and 3R and 7R formed separate groups. Again, the GD matrices based on total and NA markers formed separate groups.

An analysis of the Mantel test showed that the genetic distance matrices of SNPs and silicoDArTs evaluated for all the analyzed plant materials were significantly correlated (*r* = 0.874; *p* < 0.001). However, the correlation between GD matrices of rye chromosomes varied with the marker system used for the analysis and was relatively low for SNPs (from *r* = 0.262 between 3 and 7R, to *r* = 0.383 between 1 and 4R) and somewhat higher for silicoDArTs (from *r* = 0.536 between 3 and 7R, to *r* = 0.676 between 1 and 4R) (Table 2). No matter what marker system was used, all distance matrices from individual chromosomes were very similar to those from a whole set of markers and not just assigned ones. The 1R-based GD matrices exhibited the highest correlation values with most other chromosome-based matrices. Furthermore, the 4R GD matrix evaluated based on silicoDArTs was highly correlated with the 5R and 6R, whereas the 5R matrix was highly correlated with the 6R and 2R. The SNP-based markers also showed a similar pattern, but it was not as clear.

**Table 2:** Correlations of genetic distance matrices obtained with total pool and chromosome-specific marker sets (*p* = 0.001)

		Total	1R	2R	3R	4R	5R	6R	7R
SNP	1R	0.648							
	2R	0.663	0.348						
	3R	0.603	0.300	0.281					
	4R	0.649	0.383	0.330	0.277				
	5R	0.663	0.363	0.361	0.307	0.361			
	6R	0.637	0.366	0.325	0.280	0.345	0.357		
	7R	0.652	0.329	0.316	0.262	0.318	0.346	0.340	
	NA	0.940	0.600	0.588	0.522	0.623	0.591	0.590	0.606
silicoDArT	1R	0.779							
	2R	0.729	0.631						
	3R	0.697	0.613	0.549					
	4R	0.780	0.676	0.608	0.583				
	5R	0.746	0.646	0.605	0.605	0.636			
	6R	0.765	0.671	0.606	0.587	0.645	0.645		
	7R	0.717	0.611	0.553	0.536	0.599	0.602	0.608	
	NA	0.992	0.765	0.718	0.689	0.786	0.738	0.766	0.719

AMOVA analysis revealed that RF and CMS breeding materials had a much higher proportion of within-variation (93%) than RF and CMS breeding materials (7%). The *F*_*ST*_*/Phi*_*PT*_ values between RF and CMS lines using the total number of SNP and silicoDArT markers were 0.074 (*p* < 0.001) and 0.077 (*p* < 0.001), respectively (Table 3). The same parameter varied when chromosome-assigned markers were used. Then the *F*_*ST*_ values ranged from 0.058 for 5R to 0.074 for 4R. But when silicoDArT markers were used, the *Phi*_*PT*_ values were between 0.064 (1R) and 0.082 (3R and 4R). Nei’s minimum distance (Table 3) showed a similar pattern of difference between RF and CMS lines.

**Table 3:** Fixation index (*F*_*ST*_), pairwise values (*Phi*_*PT*_) and Nei’s minimum genetic distance between restorer and cytoplasmic male-sterile lines included in the Polish hybrid breeding program

	SNP		silicoDArT	
	*F*_ST_*	Nei’s genetic distance	*Phi*_PT_*	Nei’s genetic distance
Total	0.074	0.050	0.077	0.030
1R	0.072	0.052	0.074	0.040
2R	0.065	0.049	0.069	0.037
3R	0.072	0.052	0.082	0.043
4R	0.074	0.055	0.082	0.044
5R	0.058	0.044	0.064	0.034
6R	0.072	0.055	0.075	0.040
7R	0.070	0.052	0.073	0.040
NA	0.067	0.051	0.083	0.021

^*^F_*ST*_ is a statistic measure for comparison between codominant allelic data (SNP) and *Phi*_*PT*_ is an analogous measure for binary data (silicoDArT)

### Population structure

Principal component analysis of 94 RF and 94 CMS rye genotypes provides an estimate of the proportion explained by individual principal components (PCs) and served in our study to get a first-hand visualization of the genetic diversity and architecture. The PCA based on the total number of SNP markers shows that the first and second PCs explained only 7.8% and 4.0% of the total genetic variability of the original data set, respectively (Fig. S1). The grouping of genotypes based on total and not assigned SNP markers is similar. It reveals that at least two separate subgroups exist. However, 17 of the RF lines are located in a subgroup represented by mostly CMS lines, and 15 of the CMS lines are located in the RF subgroup. Data on individual chromosomes need to be clarified to determine how to divide them into subgroups (Fig. S1); however, such division is not excluded in some cases.

The PCA done on all of the silicoDArT markers put the 188 rye accessions into two main groups, which were made up of the RF and CMS lines (Fig. S2). The first and second principal components explained 5.8% and 3.0% of genetic variability, respectively. When the pool of unassigned markers was used in the analysis, it showed the same grouping. The only exceptions are the restorer line (“L1409”), which shared ancestry with male-sterile genotypes, and the CMS line (S133P), which was grouped with the RF materials. When markers assigned to individual chromosomes were applied for the PCA, the division into two groups was still present (Fig. S2). However, the differentiation of the RF and CMS materials was somewhat unclear, with apparent regions where representatives of the CMS and RF pools are mixed. Furthermore, variation in RF and CMS populations was observed depending on the chromosome-assigned markers used. For example, when 1R data is used, CMS line variation is much higher than RF; when 4R data is used, it is the other way around.

**Fig. 4:**
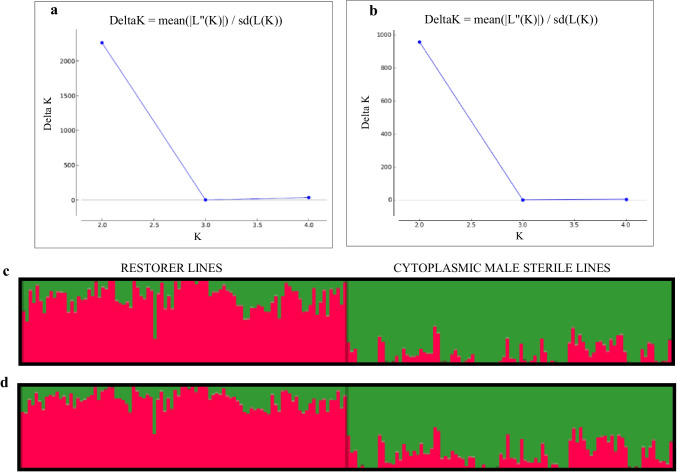
Determination of the optimal value of K = 2 and population structure analysis of 94 restorer lines (RF) and cytoplasmic male-sterile (CMS) lines using silicoDArT (**a**) and SNP (**b**) data. Illustration of population structures evaluated on the total pool of SNP (**c**) and silicoDArT markers (**d**)

Based on the total number of SNPs or silicoDArTs, an analysis of the population structure showed that the data was split into two groups (P1 and P2). The highest ΔK value was observed at *K* = 2 (Fig. 4a and b). SNP markers revealed more vital structuring than silicoDArTs (ΔK = 1961.5 for SNPs; ΔK = 959.3 for silicoDArTs). Among all the accessions tested using SNP and silicoDArT markers, 93 restorer lines formed one population (Table 4, Table S1). In contrast, the remaining lines belonging to the CMS pool formed the second population (Fig. 4c and d). It should be stressed, however, that the CMS pool encompasses a single restorer line (L1409). Due to only minor deviations, the results of AMOVA for a structured population based on total pools of markers were the same as for RF and CMS pools (not shown).

**Table 4:** The arrangement of structure results and “misassignment” of RF and CMS lines depending on marker type and their chromosomal location

		1R	2R	3R	4R	5R	6R	7R
SNP	K	2	2	2	2	2	2	3*
	ΔK value	318.5	261.6	149.1	216.8	399.7	204.6	477.2
	Number of **RF** lines in **P1**	66 (70.2%)	51 (54.3%)	72 (76.6%)	70 (74.5%)	70 (74.5%)	93 (98.9%)	49 (52.1%)
	Number of **CMS** lines in **P2**	93 (98.9%)	94 (100%)	90 (95.7%)	94 (100%)	83 (88.3%)	89 (94.7%)	94 (100%)
	Number of RF lines in P2	28	43	22	14	14	1	23
	Number of CMS lines in P1	1	0	4	0	11	5	0
silicoDArT	K	2	2	2	2	2	2	3*
	ΔK value	468.6	490.7	428.2	1329.9	323.3	354.6	501.8
	Number of **RF** lines in **P1**	89 (94.7%)	92 (97.9%)	86 (91.5%)	94 (100%)	92 (97.9%)	92 (97.9%)	40 (42.5%)
	Number of **CMS** lines in **P2**	92 (97.9%)	90 (95.7%)	78 (82.9%)	88 (93.6%)	86 (91.5%)	94 (100%)	94 (100%)
	Number of RF lines in P2	5	2	8	0	2	2	30
	Number of CMS lines in P1	2	4	16	6	8	2	0

^*^The rest of the RF lines represented P3

The percentage of RF and CMS lines in a given population (P1 or P2) is placed next to the numerical values

The markers associated with the rye chromosomes generally assessed a similar number of populations (Table 4, Table S1). In the case of the 1R-6R chromosomes, *K* equals two. However, for the 7R chromosome, *K* equals three regardless of whether SNPs or silicoDArTs were employed. Chromosome-assigned silicoDArT markers (in contrast to the entire marker pool) revealed more robust plant material structuring than SNPs (Table 4). Based on chromosome-specific SNPs or silicoDArTs, the analyzed plant samples were not precisely put into one of the populations in the same way as was done with the whole marker pool. Using SNP markers, the correct classification of RF lines to P1 ranged from 52.1% for 7R to 98.9% for 6R, and silico-DArTs from 42.5% (7R) to 100% (4R). A similar analysis shows that most CMS lines are classified as P2. The percentage values varied from 88.3% (5R) to 100% (2R, 4R, and 7R) for SNPs and from 82.9% (3R) to 100% (6R and 7R) for silicoDArTs. Regarding 7R-located markers, SNPs and silicoDarTs group the CMS-based lines into one population and divide the restorers into three populations.

## Discussion

Genetic differentiation of breeding populations originating from distinct sources may indicate the formation of heterogenic pools (Vogt et al. 2020). Combined, they may result in extra heterozygosity, increasing yield, tolerance to abiotic stresses (Anioł and Gustafson 1984; Matos 2005), and confer extra resistance to pathogens (Miedaner et al. 2002). In the Polish breeding populations of rye, such pools were hardly recognized, mainly due to common breeding programs leading to the exchange of materials (Bolibok-Brągoszewska et al. 2014). However, with the development of the CMS Pampa hybrid approach (Geiger and Schnell 1970) and its introduction by Lucjan Madej (Madej 1975), Polish companies started separating their materials. Some specialized in maternal, whereas the others in parental forms used as components of new cultivars. A good example is the “Gulden” cultivar (DANKO Plant Breeding Co. Ltd) released recently (COBORU 2022a) or those made available to farmers years ago (Arseniuk and Oleksiak 2003). Still, Polish rye hybrids’ yield is lower than those developed in Germany (COBORU 2022b). The reason for that is the preference for 1R rather than 4R restorer QTL in Polish materials. The frequency of the 4R QTL is relatively minute. However, in some cases, such materials were identified (Niedziela et al. 2021a).

The selection of hybrid materials via a breeding approach is time-consuming. Thus, alternative methods based on molecular markers are needed. While the genomic selection approach is of the highest importance and gave acceptable results in wheat (Michel et al. 2017; Zhao et al. 2021), corn (Zhang et al. 2017a, b), and many other kinds of cereal (Cui et al. 2020; Wolfe et al. 2017), it was not tested for rye. Still, the limited pool of available materials for such selection makes the approach unjustified. The other option is to make markers for pollen fertility restoration QTLs and use them for selection. While many markers linked to or associated with the QTLs were evaluated (Niedziela et al. 2021b; Stojałowski et al. 2011), few were tested on a broad range of materials. Alternatively, molecular markers combined with clustering methods could be used. But the method was questioned because the results depended on the species and the marker system (Tomkowiak et al. 2020; Dziurdziak et al. 2021). The other option is to identify the respective genetic pools and assess their suitability for hybrid breeding.

Based on PCA with a full pool of SNPs or silicoDArTs, the current study shows that plant materials from two breeding companies that run maternal or parental components of the hybrids are different. Based on the results of AMOVA, the difference between the two pools explained 7.4% (SNPs) and 7.7% (silicoDArTs) of the variance. A similar comparison of the German hybrid breeding pool showed that the *F*_*ST*_ values were at least two times higher in the cases of a broad range of rye materials (Bauer et al. 2017) and about five times higher for the RF and CMS-based materials (Vendelbo et al. 2020). The fact was interpreted in terms of genetic pool formation. The PCA analysis confirmed the difference. It shows apparent differences between the RF and CMS materials, where only a small number of lines from each group were put in the wrong group regarding the breeding company. A study of the structure of the population confirmed the result, which showed that two different genetic pools of rye materials have grown up that are used only for hybrid breeding. But the differences may not be due to the creation of pools that can be used to choose materials for hybrid breeding. Instead, they may be the result of breeding preferences. Instead of using all the markers, looking at the ones unique to each rye chromosome might be more helpful. This would help us tell where the plants came from and whether they belonged to the RF or CMS groups. As expected, AMOVA on the markers put on each rye chromosome showed that RF and CMS materials differed. According to AMOVA, the difference between RF and CMS materials depends on the chromosome and marker type.

Furthermore, the highest values of explained variance were evaluated for the 4R (SNPs) and the 4R and 5R (silicoDArTs) chromosomes. The presented result is in line with our hypothesis that breeding materials for RF and CMS pools should force selection at the level of chromosomes carrying pollen fertility restoration traits and that the differentiation of plant materials based on the markers assigned to those chromosomes should be stronger than the total number of markers of the given type. The outcome is consistent with genetic distance, PCA, and structure analyses. More exposed results were found for silicoDArTs than SNPs, showing that they are better at separating rye lines. The difference between marker systems may reflect their dominant and codominant natures. Codominant markers may identify heterozygotic loci, so they should be less distinctive than dominant markers. Alternatively, the number of SNPs and silicoDArTs may also be substantial. In general, more silicoDArTs were available for the analyses of those SNPs. However, independently of the marker type, they were evenly distributed along each rye chromosome without significant gaps. Thus, their number and distribution should not affect the results (Table S2). The differences reflect the available RF and CMS plant material differentiation.

The notion seems to be confirmed by the Tukey’s HSD test comparing genetic distances evaluated based on silicoDArTs. It was shown that 1R, 4R, and 6R formed a single group. The other chromosomes, 2R and 5R, and 3R and 7R, formed another one, suggesting that the chromosomes belonging to the same group might be under comparable pressure. In the case of the first group, the grouping may reflect pollen fertility restoration as the chromosomes are responsible for the expression of the trait. A comparable analysis conducted on SNPs failed to identify similar grouping (3R, 4R, 6R, and 7R vs 1R, 2R, and 5R). This is in agreement with the fact that distinct information could be gained depending on the marker system and type. Therefore, selecting the marker system and type should be carefully considered for hybrid breeding purposes.

The results of the GD analysis show that the lowest averages based on SNPs for 1R (0.562) and 5R (0.551) might show less polymorphism in some genomic regions where there are QTLs that control traits that are important for agriculture. The 1RS is known to carry a cluster of genes encoding resistance to stem, leaf, and yellow rust (Mago et al. 2005), as well as one of the most influential and widely used Pm genes to control powdery mildew (Wricke et al. 1996; Hsam et al. 2000; Simkova et al. 2008). In European rye resources, Meidaner et al. (2000) found that the short arm of the 1R chromosome is home to the QTL that helps CMS Pampa grow back the best. The other essential traits, including plant height, spike length, the number of florets (Plaschke et al. 1993; Börner et al. 1999), and alpha-amylase activity (Masojć and Milczarski 2009), are localized on 5R. At the same time, GD shows that despite high within-population variance (93%), some materials were very similar. The result may suggest that the RF and CMS gene pools may be limited and need to be enriched with new genotypes.

The other question we are trying to answer with our work is whether or not any grouping analysis can be used to predict materials that would fit into the maternal or paternal restoration pool. The most logical approach would be to use chromosomal markers that reflect pollen fertility and/or sterility. For example, in rye pollen fertility restoration, QTLs are present on 1R, 3R, 4R, 5R, and 6R (Miedaner et al. 2000), with the most significant on 4R originating from Iranian and Argentinian germplasm (Miedaner et al. 2000).

Focusing attention on the classification of RF and CMS lines (based on structure analysis), it could be shown that CMS lines are usually completely separated from the pool of RF materials. The highest misclassification was about 17% for SNPs (5R) and silicoDArTs (3R) chromosomes. Generally, the RF lines were inappropriately classified, even in 46% of cases utilizing SNPs (2R) and 56.5% for silicoDArTs (7R). However, misclassification is less pronounced for the 1R and 4R chromosomes, which carry the most significant pollen fertility restoration QTLs. Only 30% of the RF and about 1% of CMS materials were misclassified based on SNPs. When silicoDArTs were applied, the level of misclassification equaled 5% and 2% for the RF and CMS materials, respectively. In the case of the 4R data, up to 74.5% of the RF and 100% of the CMS lines were assigned according to the known phenotypes based on SNPs. The silicoDArTs were even more efficient in the case of RF materials (100% correct assignment), and 93.6% of the CMS lines were grouped according to expectations. Based on the markers that were assigned to the 1R and 4R chromosomes, the data could be used to separate the hybrid breeding materials into two groups. So, the result may show that materials from different companies show signs of forming heterotic pools. The idea is backed up by the fact that when markers for the other chromosomes were used, there were more wrong classifications. To confirm the notion, though, more research needs to be done on combining the abilities of the two groups.

## Conclusions

In conclusion, the choice of marker system is crucial when trying to find the genetic pools of RF and CMS materials. Dominant markers are more effective than codominant ones in separating RF and CMS-based materials. It is best to find out which chromosomes have the most to do with the trait since the markers on those chromosomes would be better at separating materials than the total number of markers. The most differentiating RF and CMS materials markers were those mapped to 1R and 4R chromosomes, which carry the most important QTLs for the trait. The two pools of plant materials derived from two companies and reflecting RF and CMS hybrid components have signs of differentiation, suggesting the opportunity for independent genetic pool formation. The presence of genetically closely related materials in the RF and CMS materials suggests the necessity of extending the genetic pool.

## Supplementary Information

Below is the link to the electronic supplementary material.

Supplementary file1 (PDF 205 KB)

Supplementary file2 (PDF 207 KB)

Supplementary file3 (XLSX 62 KB)

Supplementary file4 (XLSX 878 KB)

## Data Availability

All supporting data are included within the article and its additional files.
