# Supplementary material for: Population structure and genetic diversity of a germplasm for hybrid breeding in rye (Secale cereale L.) using high-density DArTseq-based silicoDArT and SNP markers
Source: J Appl Genet. 2023 Jan 3;64(2):217–29. doi: 10.1007/s13353-022-00740-w (PMC10076414; doi:10.1007/s13353-022-00740-w)

Fig. S2 Principal component analysis (PCA) based on silicoDArTs and Jaccard genetic distance matrixes of the rye accessions: RF-black dots; CMS - red dots.

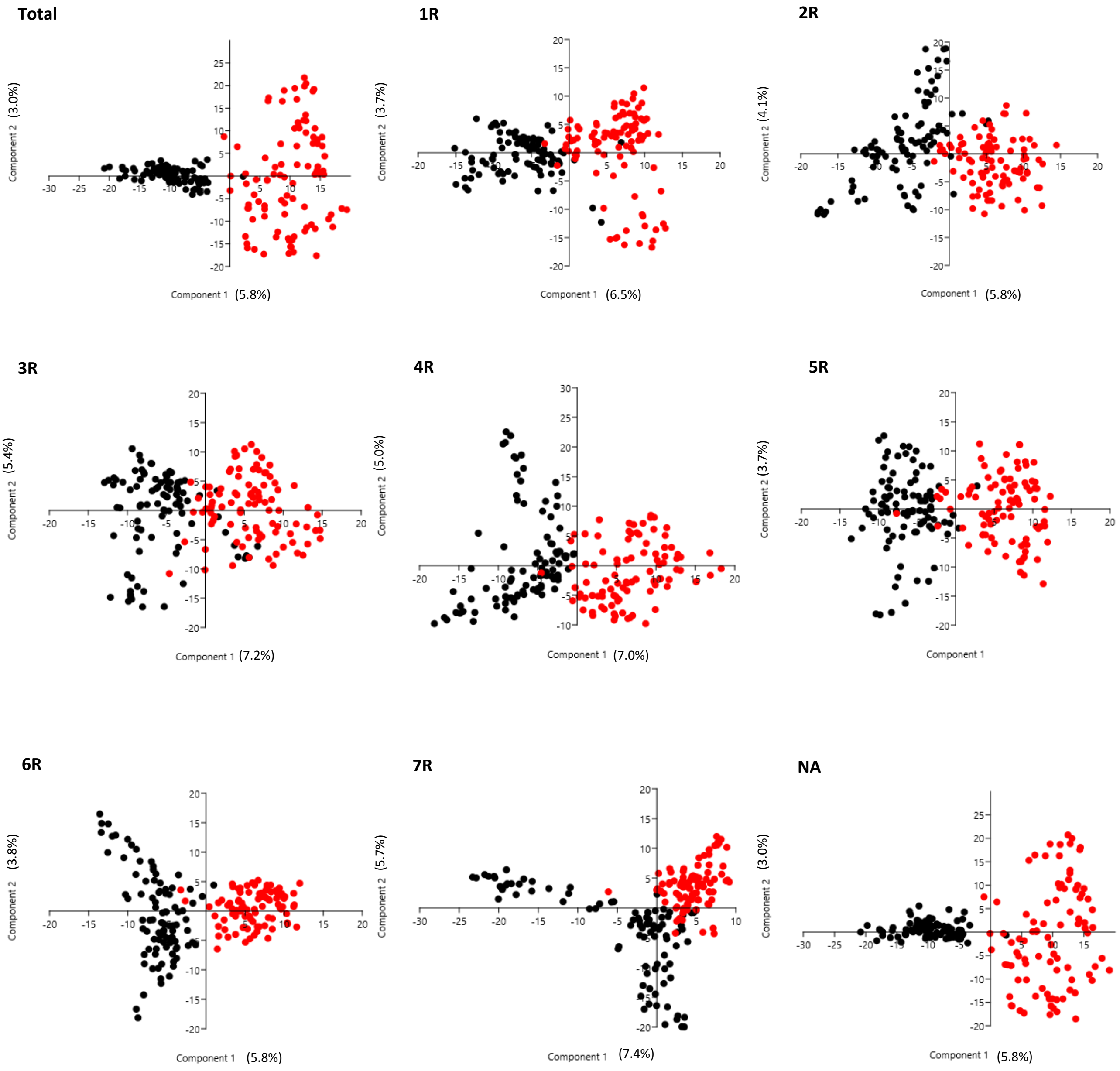

Supplement: Supplementary file 2 — Supplementary file2 (PDF 207 KB) [file 13353_2022_740_MOESM2_ESM.pdf]
